# Collagen triple helix repeat containing 1 is a new promigratory marker of arthritic pannus

**DOI:** 10.1186/s13075-016-1067-1

**Published:** 2016-07-19

**Authors:** Mohammed Talha Shekhani, Toni S. Forde, Altynai Adilbayeva, Mohamed Ramez, Askhat Myngbay, Yergali Bexeitov, Volkhard Lindner, Vyacheslav A. Adarichev

**Affiliations:** Albert Einstein College of Medicine, Departments of Medicine (Division of Rheumatology) and Microbiology & Immunology, Bronx, NY 10461 USA; National Laboratory Astana, Astana, 010000 Kazakhstan; Maine Medical Center Research Institute, Scarborough, ME 04704 USA; Department of Biology, Nazarbayev University, School of Science and Technology, Astana, 010000 Kazakhstan

**Keywords:** Rrheumatoid arthritis, Animal model, Synoviocytes, Cell motility

## Abstract

**Background:**

The formation of destructive hypercellular pannus is critical to joint damage in rheumatoid arthritis (RA). The collagen triple helix repeat containing 1 (CTHRC1) protein expressed by activated stromal cells of diverse origin has previously been implicated in tissue remodeling and carcinogenesis. We recently discovered that the synovial *Cthrc1* mRNA directly correlates with arthritis severity in mice. This study characterizes the role of CTHRC1 in arthritic pannus formation.

**Methods:**

Synovial joints of mice with collagen antibody-induced arthritis (CAIA) and human RA-fibroblast-like synoviocytes (FLS) were immunostained for CTHRC1, FLS and macrophage-specific markers. CTHRC1 levels in plasma from patients with RA were measured using sandwich ELISA. The migratory response of fibroblasts was studied with a transwell migration assay and time-lapse microscopy. Velocity and directness of cell migration was analyzed by recording the trajectories of cells treated with rhCTHRC1.

**Results:**

Immunohistochemical analysis of normal and inflamed synovium revealed highly inducible expression of CTHRC1 in arthritis (10.9-fold). At the tissue level, CTHRC1-expressing cells occupied the same niche as large fibroblast-like cells positive for α-smooth muscle actin (α-SMA) and cadherin 11 (CDH11). CTHRC1 was produced by activated FLS predominantly located at the synovial intimal lining and at the bone-pannus interface. Cultured RA-FLS expressed CDH11, α-SMA, and CTHRC1. Upon treatment with exogenous rhCTHRC1, embryonic fibroblasts and RA-FLS significantly increased migration velocity, directness, and cell length along the front-tail axis (1.4-fold, *p* < 0.01).

**Conclusion:**

CTHRC1 was established as a novel marker of activated synoviocytes in murine experimental arthritis and RA. The pro-migratory effect of CTHRC1 on synoviocytes is considered one of the mechanisms promoting hypercellularity of the arthritic pannus.

**Electronic supplementary material:**

The online version of this article (doi:10.1186/s13075-016-1067-1) contains supplementary material, which is available to authorized users.

## Background

Hypertrophy of the synovial membrane accompanied by increased production of cartilage-degrading and bone-degrading enzymes is a hallmark of arthritis. Triggered by local inflammation in the synovial joint, normal fibroblast-like synoviocytes (FLS) are substituted with newly formed hypercellular granulation tissue, the pannus. Hypercellularity of the pannus could be explained by the proliferation of synovial intimal lining cells, but the pannus is largely formed by immigration into the synovium hematopoietic and mesenchymal precursors. The latter cell lineage gives rise to the phenotype of activated FLS [1, 2].

In addition to the increased proliferation and production of a wide spectrum of cartilage-degrading proteases, activated FLS demonstrate a high level of invasiveness and motility within the affected joint, and FLS are even capable of spreading the disease to as yet unaffected cartilage using vasculature-independent routes [3]. Invasive properties of activated FLS from patients with rheumatoid arthritis (RA) and from murine arthritis models directly correlate with the rate of joint destruction and deformities, and poor disease outcome [4]. In that regard, FLS are similar to invasive tumor cells: FLS accumulate somatic mutations in the p53 tumor suppressor gene and acquire an activated expression status of several oncogenes that altogether contribute to the pathological FLS phenotype [2, 5].

Activated FLS express a number of markers, but none of them alone could be called completely specific for FLS. One of the relatively unique surface markers for RA-FLS is cadherin-11 (CDH11), which plays an essential role in the acquisition of the invasive phenotype of activated FLS in arthritis [2, 6]. However, CDH11 is expressed in many other mesenchymal-type cells and is involved in the regulation of invasiveness of, for instance, prostate and glioblastoma tumors, and also promotes bone metastasis [7, 8].

Recently, we found that the expression of collagen triple helix repeat containing 1 (CTHRC1) encoding gene was strongly associated with the severity of murine collagen antibody-induced arthritis (CAIA) [9]. When CAIA is genetically attenuated by the *Pgia8* locus in BALB/c.DBA/2-*Pgia8* congenic mice, expression of *Cthrc1* is decreased along with expression of mRNAs of metalloproteinase *Adamts12* and wingless (WNT)-associated pathway members r-spondyn 2 and syndecan 2.

CTHRC1 protein is expressed in a number of embryonic and neonate tissues, including developing cartilage and bone [10]. Experiments with gene-deficient and transgenic mice indicate that CTHRC1 regulates osteoblastic bone formation [11]. CTHRC1 can inhibit Smad2/3 phosphorylation after stimulation by transforming growth factor (TGF)-ß and can reduce production of collagen types I and III [12]. Overexpression of *Cthrc1* in smooth muscle cells and embryonic fibroblasts correlates with increased cell migration properties [13]. The endogenous expression of CTHRC1 has been found in more than a dozen types of metastatic solid cancer, and the inhibition of CTHRC1 expression results in decreased cell migration in vitro [14]. Immunohistochemical analysis of various human primary cancers and metastases has revealed that CTHRC1 expression is actually limited to the stromal cells of solid tumors [15, 16]. In this study, we analyzed CTHRC1 expression in synovium and established this protein as a novel marker of enhanced migratory potential of fibroblast-like cells, including activated FLS.

## Methods

### Patients

Biological samples were obtained under a protocol approved by the Institutional Research Ethics Committee (IREC) of Nazarbayev University, Astana, Kazakhstan. All subjects gave written informed consent. Patients coming to outpatient facility of the Republican Diagnostics Centre (RDC, Astana, Kazakhstan), who presented with clinically apparent synovial swelling were examined for RA symptoms. The final diagnostic outcome was based on persistent inflammatory arthritis, magnetic resonance imaging (MRI), radiographic analysis, disease duration, and number of tender and swollen joints, and resulted in the 28-joint-count disease activity score (DAS28). Clinical assessment was accompanied with peripheral blood analysis for complete blood counts with differential, C-reactive protein (CRP), erythrocyte sedimentation rate (ESR), rheumatoid factor (RF), and anti-citrullinated protein antibodies (ACPA). Venous blood was collected into heparinized tubes, and cells were removed by centrifugation at 1000 × g for 10 minutes. Plasma was stored at –80 °C.

### ELISA for CTHRC1

Sandwich ELISA was used to quantify CTHRC1 in human plasma according to the manufacturer’s protocol (www.mmcri.org/antibody, Maine Medical Center Research Institute, Scarborough, ME, USA). Briefly, 96-well plates (Maxisorp, Nunc) were coated overnight at 4 °C with capture antibody 13E09 at 1.8 μg/ml in carbonate-bicarbonate buffer pH 9.4. All subsequent procedures were performed at room temperature. The next day, wells were washed twice with PBS containing 0.1 % BSA and 0.1 % Tween 20 (buffer PBS-BT) and then blocked with PBS-BT for 1 hour. Human plasma or conditioned media were diluted at least 1:5 in PBS-BT and incubated with absorbed capture antibodies for 2 hours. Subsequently, the wells were washed and then incubated with biotinylated detection antibody Vli10G07 diluted 1:500 in PBS-BT for 1 hour. After washing, wells were treated for 1 hour with streptavidin conjugated with horseradish peroxidase (St-HRP) (Pierce High Sensitivity St-HRP, Thermo Fisher Scientific Inc., Waltham, MA, USA) diluted at 1:8,000 in PBS-BT. After the final wash, TMB (3,3′,5,5′-tetramethylbenzidine) chromogenic substrate (Amresco, Solon, OH, USA) was added, and the developed signal was measured at 450 nm using the Multiscan-FC plate reader (Thermo Fisher Scientific Inc., Waltham, MA, USA). Absorbance was converted to absolute concentration using rhCTHRC1 as a reference. ELISA was performed in triplicates.

### Animals and arthritis induction

Mice were housed in a specific pathogen-free environment in the Institute for Animal Studies at the Albert Einstein College of Medicine, Bronx, NY, USA. All animal experiments were approved by the Institutional Animal Care and Use Committee (IACUC) of the Albert Einstein College of Medicine. Inbred BALB/c female mice, 2–4 months old (Charles River, Wilmington, MA, USA) were injected with ArthritoMab antibody cocktail (MD Biosciences Inc., St.Paul, MN, USA) according to the manufacturer’s protocol. Briefly, mice were injected intraperitoneally (i.p.) with 2 mg/mouse of antibodies on day 0 followed by i.p. injection of 40 μg of lipopolysaccharide (LPS) on day 3. Arthritis in the (FVB/N × BALB/c) F1 hybrid mice was induced similarly, but using CIA-MAB-2C ArthritoMab Cocktail (MD Biosciences Inc.), which was developed for murine strains that are refractory to arthritis. Mice were observed once or twice a day for paw swelling and redness by two independent observers. The arthritis scoring system was based on the number of inflamed joints in each paw producing a total score of 0–60 per mouse, as described previously [9, 17].

### Real-time PCR

Total RNA was isolated from mouse paws using the RNeasy Mini kit (Qiagen Inc., Valencia, CA, USA) according to the manufacturer’s instructions. Paw tissue was taken from digits to the ankle or to the distal radius/ulna. RNA integrity and quality was tested on an Agilent 2100 Bioanalyzer. The Superscript First-Strand Synthesis kit (Invitrogen Life Technologies, Grand Island, NY, USA) was used to generate cDNA that was assayed using an Applied Biosystems 7900HT Fast Real-Time PCR System. Expression was measured in triplicates and at least two repeats using the Fast SYBR Green system (Applied Biosystems by Life Technologies, Grand Island, NY, USA). *Cthrc1*-specific primers were forward 5′-GGGATGGATTCAAAGGGGAAA-3′ and reverse 5′-AGAACTCGCAGAGCACTGTT-3′. *Gapdh*-specific primers were forward 5′-ACCCAGAAGACTGTGGATGG-3′ and reverse 5′-ACACATTGGGGGTAGGAACA-3′. Relative RNA concentration was calculated using the delta-delta cycle threshold (Ct) method corrected for PCR efficiency, as described previously [9, 18].

### Cell lines

Human RA-FLS derived from the inflamed synovial tissue of patients with RA (Cell Applications Inc., San Diego, CA, USA) were used at passages three to four. RA-FLS were maintained in Dulbecco’s modified Eagle’s medium (DMEM) with GlutaMAX-1, 4.5 g/l D-glucose, and 25 mM HEPES, antibiotic and antimycotic (Gibco by Life Technologies, Grand Island, NY, USA) supplemented with 10 % heat-inactivated FBS and growth supplements (Cell Applications Inc., San Diego, CA, USA). Mouse embryonic fibroblast cell lines C3H/10T1/2 and NIH 3T3, and human skin fibroblast line Hs68 (all lines from ATCC Inc., Manassas, VA, USA) were studied.

### Histopathology and immunohistochemical analysis (IHC)

Mice were killed by CO_2_ inhalation according to the approved IACUC protocol. For histopathological analysis, hind limbs from the digits to femur, including the knee joint, were collected. Tissues were fixed in 10 % neutral buffered formalin, decalcified using Immunocal reagent (Decal Chemical Corporation, Suffern, NY, USA) and embedded in paraffin. Tissues were sectioned at a thickness of 5 μm and stained with hematoxylin-eosin or alcian blue and nuclear fast red for histological confirmation of arthritis.

For immunohistochemical analysis, sections were deparaffinized and pretreated with 10 mM sodium citrate buffer at pH 6.0 heated to 96 °C for 20 minutes for antigen retrieval. Endogenous peroxidase activity was quenched by incubation in 0.3 % hydrogen peroxide in PBS for 5 minutes. Nonspecific binding was blocked for 1 hour at room temperature using 2 % BSA (Sigma-Aldrich, St. Louis, MO, USA) and 2 % normal goat serum (Vector laboratories Inc., Burlingame, CA, USA) in PBS and 0.05 % Tween-20 (PBS-T buffer). For detection of FLS-specific and macrophage-specific markers in arthritic pannus, we used rabbit anti-CTHRC1 antibodies (Vli-55, www.mmcri.org/antibody, Scarborough, ME, USA), rabbit anti-IBA1 antibodies (#019-19741, Wako Chemicals USA Inc., Richmond, VA, USA), rabbit anti-CDH11 antibodies (WTID1, Invitrogen by Life Technologies, Grand Island, NY, USA), and rabbit antibodies to smooth muscle actin (α-SMA) (E184, Abcam, Cambridge, MA, USA). As a secondary antibody, donkey anti-rabbit antibodies conjugated to HRP (Abcam, Cambridge, MA, USA) were used. After three washes with PBST, slides were finally stained with ImmPact 3,3′-diaminobenzidine (DAB) and counterstained with hematoxylin (Vector laboratories Inc.) or alcian blue (Thermo Fisher Scientific Inc., Waltham, MA, USA). Sections were dehydrated and mounted with Permount (Thermo Fisher Scientific Inc.).

### Recombinant protein overexpression

We used a full-length human *Cthrc1* cDNA cloned in frame with turbo-GFP (clone RG204348, OriGene Technologies, Inc., Rockville, MD, USA) as a template to amplify a *Cthrc1* sequence. High fidelity PCR using Herculase II fusion DNA polymerase (Agilent Technologies, Inc., Santa Clara, CA, USA) with forward 5′-ATTTGGTACC**ATG**CGACCCCAGGGCCCCGCCG-3′ and reverse 5′-AATAGCGGCCG**CTA***ATGATGATGATGATGATG*TTTTGGTAGTTCTTCAAT-3′ primers/linkers to introduce sites for restriction endonucleases Kpn°I and Hind°III and 6xHis tag and stop codon (restriction sites are underlined; ATG and stop codons are boldfaced; 6xHis coding sequence is italicized and underlined) for cloning the amplicon into the pCMV6-K/N vector (OriGene Technologies, Inc.). The recombinant construct structure was confirmed with a complete sequencing (Thermo Fisher Scientific Inc., Fair Lawn, NJ, USA).

Transient transfection of the Chinese hamster ovary (CHO) cells with lipofectamine LFA2000 was performed according to the manufacturer’s protocol (Life Technologies, Grand Island, NY, USA). CHO cells were grown in Opti-CHO chemically-defined serum-free conditioned media Opti-CHO (Gibco Life Technologies, Grand Island, NY, USA) for 5 days. Conditioned media were collected and centrifuged at 1000 × g for 15 minutes to separate from floating cells.

### Western immunoblotting

Cells were lysed in Laemmli sample buffer (62.5 mM Tris-HCl, 2 % sodium dodecyl sulfate (SDS), 10 % glycerol, 0.05 % bromphenol blue, and 100 mM 2-mercaptoethanol). Whole cell/tissue lysates were separated on 4–15 % gradient SDS-polyacrylamide gels (Bio-Rad Laboratories Inc., Hercules, CA, USA) and transferred onto polyvinylidene fluoride membrane (Thermo Fisher Scientific Inc., Waltham, MA, USA). Membranes were blocked for 1 hour in 1 % (w/v) casein in 25 mM Tris pH 7.4, 150 mM NaCl, 0.1 % Tween 20 (TBS-T) (casein blocking buffer, Thermo Fisher Scientific Inc.). After blocking, the membranes were probed with rabbit antibodies to CTHRC1 (Vli-55, www.mmcri.org/antibody, Scarborough, ME, USA), rabbit antibodies to CDH11 (Invitrogen Life Technologies, Grand Island, NY, USA), rabbit antibodies to α-SMA (Abcam, Cambridge, MA, USA), mouse antibodies to α-tubulin (Sigma-Aldrich, St. Louis, MO, USA) as protein load control. As secondary antibodies, HRP-conjugated donkey anti-rabbit, anti-mouse or anti-sheep antibodies (Abcam) were used. Enhanced chemiluminescent substrate (Thermo Fisher Scientific Inc.) and HyBlot-CL film (Denville Scientific Inc., Holliston, MA, USA) were used to detect a signal. Optical density was quantified by ImageJ analysis software [19].

### Cell migration assay

Cell migration was assessed using transwell permeable supports with an 8-μm pore membrane (Corning Costar, Tewksbury, MA, USA). C3H/10 T1/2 murine embryonic fibroblasts or RA-FLS were seeded in the upper chamber in DMEM supplemented with 1 % BSA. The lower chamber was filled with DMEM containing 0 %, 1 %, 6 %, or 10 % FBS. rhCTHRC1 (Sino Biological Inc., Beijing, China) or rhCTHRC1 (OriGene Technologies Inc., Rockville, MD, USA) were added to the upper chamber at 500–2,000 ng/ml. After 18 hours at 37 °C in 5 % CO_2_, cells were fixed with 0.3 % glutaraldehyde in PBS for 10 minutes at room temperature and then stained with toluidine blue. Upper surface of the membrane was cleaned to remove any non-migrated cells using a cotton swab. Membranes were peeled off, and mounted onto glass slides for microscopic examination. Transmigrated cells were counted at 200 × magnification in four to eight fields of view using bright field microscopy.

### Time-lapse microscopy

Murine NIH 3T3 fibroblasts were plated on collagen type I-coated μ-slides (IBIDI USA Inc., Madison, WI) and incubated for 4 hours at 37 °C in 5 % CO_2_/95 % air in complete DMEM media supplemented with 10 % FBS to insure complete cell adhesion on a bridge. Subsequently, rhCTHRC1 protein (Sino Biological Inc., Beijing, China) was added at 1000 ng/ml, and imaging has been performed for 14 hours in a serum-containing medium at 37 °C using time-lapse phase contrast microscopy with a Cell Observer microscope (Carl Zeiss Microscopy GmbH, Göttingen, Germany). Recorded stacks of images were transferred to ImageJ [19] and further analyzed with the MTrackJ plugin and Chemotaxis Tool (IBIDI USA Inc., Madison, WI, USA). The trajectories of individual cells were evaluated for accumulated distance migrated (Ad) and for cell velocity. Directness was calculated as a ratio of Euclidean distance (Ed) to the accumulated distance (Ed/Ad) (Fig. 6). Additionally, elongated morphology cell shape was studied (horizontal polarization), which was defined as maximal cell dimension using linear approximation. In preliminary experiments, we found that pro-motile and polarization effects of the CTHRC1 treatment were pronounced in the time window from 4–12 hours, and thereafter all calculations were performed for this time period.

### Statistical analysis

Data were analyzed using Student’s *t* test for unpaired samples, using SPSS statistical software (SPSS Inc., Chicago, IL, USA). The Mann–Whitney *U* test for unpaired samples was performed using GraphPad Prism6 software (GraphPad Software Inc., La Jolla, CA, USA). *P*-values less than 0.05 were considered significant. Quantitative measurement of brown-colored DAB product of IHC staining was performed using ImageJ software [19] by color channel separation and densitometry of microscopic images at × 200–400 magnification. On each slide, at least five positively stained areas were analyzed for integrated brown-colored density; at least five slides per mouse (n = 5 per group) were analyzed.

## Results

### CTHRC1 expression is strongly inducible in murine arthritis

**Fig. 1:**
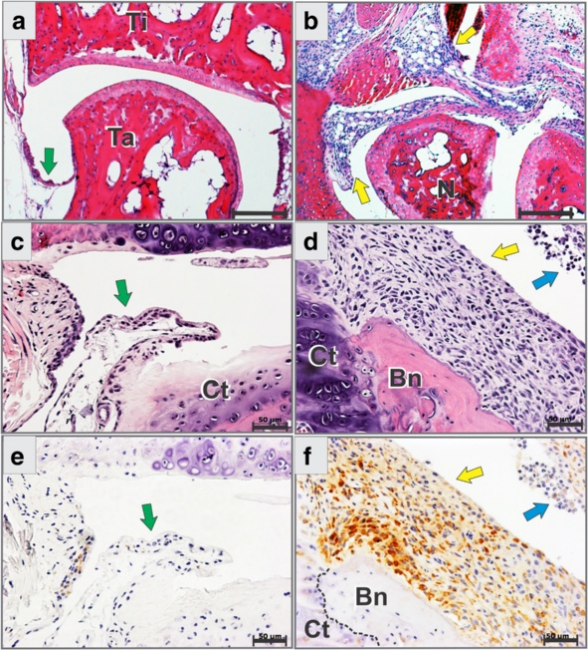
Collagen triple helix repeat containing 1 (CTHRC1) expression is inducible in arthritis. Histology of hind limb (**a**, **b**) and knee joint (**c**–**f**) sections of naïve and arthritic BALB/c mice. *Ti* tibia, *Ta* talus, *N* navicular bone of hind paw. In naïve mice, synovial membrane is normal (**a**, **c**, *green arrowheads*). In mice with experimentally induced arthritis at the seventh day post injection of anti-collagen antibodies, massive multicellular pannus (*yellow arrowhead*) and influx of granulocytes into synovial cavity (*blue arrowhead*) were prominent (**b**, **d**). Immunohistochemical staining for CTHRC1 was negative in the normal synovial membrane (**e**) in contrast to strong positive staining of the arthritic hyperplastic synovium (**f**, *brown* 3,3′-diaminobenzidine precipitate). The border between bone (*Bn*) and cartilage (*Ct*) is shown with a *dashed line* (**f**). Sections were counterstained with hematoxylin (**e**, **f**) or stained with hematoxylin-eosin (**a**–**d**). *Scale bars* are 100 μm (**a**, **b**) and 50 μm (**c**–**f**). The data and images are representative of the two independent experiments using BALB/c wildtype inbred mice and mice from (FVB/J × BALB/c)F1 hybrid genetic backgrounds

Earlier we reported low levels of *Cthrc1* mRNA in the paws of naïve mice, but substantial upregulation of mRNA during inflammation [9]. To characterize which tissue express CTHRC1, we used IHC staining of synovial joints. We induced arthritis in BALB/c mice using injections of anti-collagen antibodies followed by LPS injections 3 days later. Using a standard protocol, we induced strong inflammation in the paws of inbred BALB/c mice and also in (FVB/N × BALB/c)F1 hybrids that are less susceptible to CAIA. Arthritis was observed in the form of swelling and redness of the fore and hind paws of mice, and the diagnosis was confirmed using histopathological analysis of hematoxylin-eosin-stained tissue sections (Fig. 1). A thin synovial membrane in naïve joints (Fig. 1a, c, green arrowheads) turned into a massively hypercellular pannus infiltrated with inflammatory cells (Fig. 1b, d, yellow arrowheads). Polymorphonuclear leukocytes were most abundant within the synovial cavity and at the side of the pannus that was facing the cavity (Fig. 1d, blue arrowheads), while pannus consisted of fibrous-like large cells in contact with the bone surface. Although the edema and redness was not visually observable in larger joints, like the knee joint, the histopathological examination showed the presence of hyperplastic synovium and leukocyte infiltration.

IHC staining of the naïve synovial joints of hind paws and knees was negative (Fig. 1e), while the amount of CTHRC1 was dramatically increased in arthritis (Fig. 1f). We studied mice with early arthritis that had been present for 2 days (5 days after mAb cocktail injection or 2 days post-LPS injection) and also in mice that had developed arthritis for 2 weeks. Joints of mice with early and 2-week inflammation were equally positive for the amount of CTHRC1 (Additional file 1). Protein was predominantly located in the hyperplastic pannus at the bone/cartilage surface (Fig. 1f, yellow arrowheads).

**Fig. 2:**
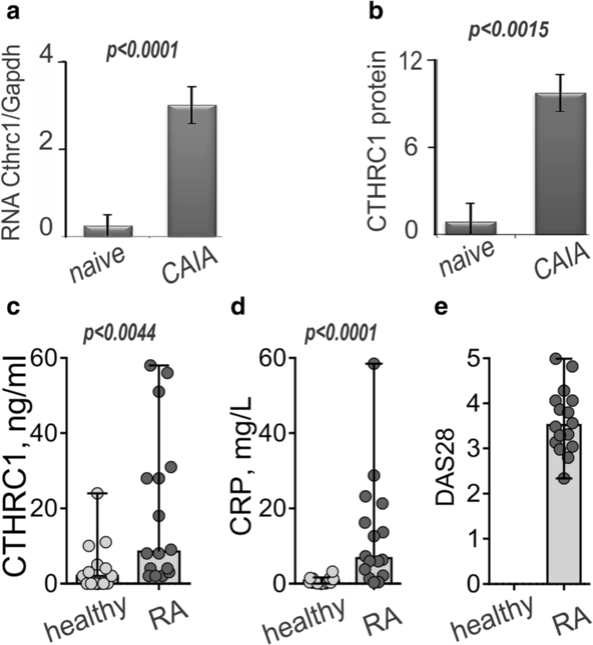
Collagen triple helix repeat containing 1 (*CTHRC1*) expression is inducible both in mouse and in human arthritis. Naïve and arthritic mouse paws were analyzed for *Cthrc1* RNA message using RT-PCR of the total RNA isolated from paws of naïve mice and mice with collagen antibody-induced arthritis (*CAIA*) (**a**). Protein content in synovium was measured using ImageJ densitometry of immunohistochemical-stained histological sections (**b**). Student’s *t* test was used to estimate the difference between arthritic and naive groups. In patients with rheumatoid arthritis (*RA*) with an average 28-joint-count disease activity score of 3.61 (**e**), blood plasma contained a significantly higher absolute concentration of CTHRC1 (**c**) and C-reactive protein (CRP) (**d**) according to the Mann–Whitney *U* test. *Horizontal lines* show median with range. *Gapdh* glyceraldehyde 3-phosphate dehydrogenase

The inducible pattern of the gene expression was corroborated at the levels of RNA and protein. At the second day after LPS injection we observed acute arthritis in mice, and *Cthrc1* mRNA was induced 11.9-fold (*p* < 0.0001), while the corresponding protein increased 10.9-fold (*p* < 0.0018) (Fig. 2a, b). Western immunoblotting of the total protein from naïve and arthritic paws confirmed that immune staining was specific for the CTHRC1 polypeptide of the predicted size (data not shown).

### Peripheral blood of patients with RA contains high levels of CTHRC1 protein

To corroborate the results based on murine arthritis, and to show evidence for a possible role of CTHRC1 in human RA, we collected clinical samples of blood plasma from healthy individuals and from patients with a diagnosis of RA. The average DAS28 in patients with RA was 3.61 ± 0.72 (average ± standard deviation), and the CRP concentration was 12.99 ± 14.91 mg/L (Fig. 2d, e). In healthy individuals, CRP was significantly lower (0.75 ± 0.86 mg/L) than in patients with RA (*p* < 0.0001, Mann–Whitney *U* test). Circulating CTHRC1 was measured in both healthy and RA cohorts (median 2.00 versus 8.50, respectively, *p* < 0.0044, Mann–Whitney *U* test) (Fig. 2c). Higher CTHRC1 in human plasma correlated with increased CRP and stronger DAS28. In both the murine arthritis model and in human arthritis, CTHRC1 protein was highly expressed compared to much lower expression in normal/basal conditions.

### Cells expressing CTHRC1 are located more frequently at the pannus boundaries

**Fig. 3:**
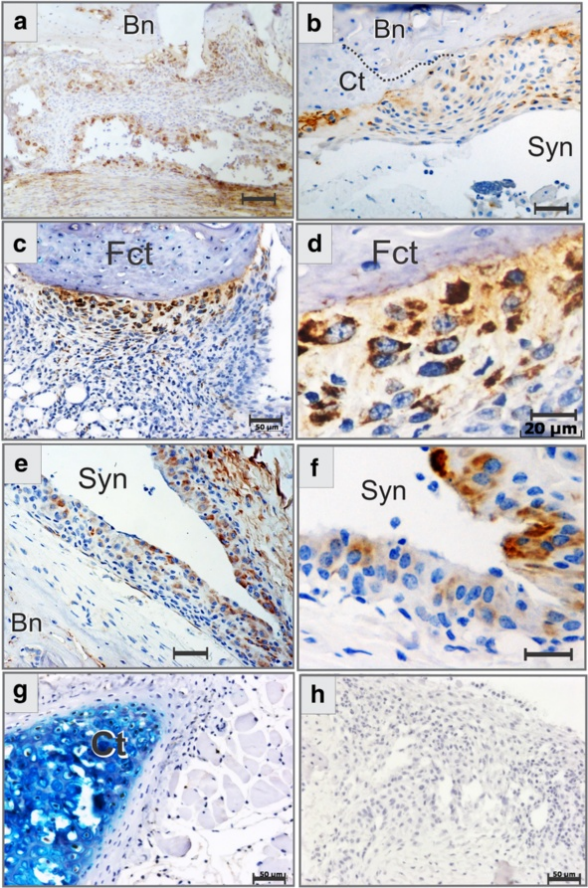
Immunohistochemical localization of collagen triple helix repeat containing 1 (CTHRC1) expressing cells in arthritic synovium. Immunohistochemical staining (IHC) for CTHRC1 was positive for hypercellular pannus of the synovial joints of the hind paws (**a**) and of the knee (**b**–**f**) of BALB/c mice with experimentally induced arthritis. Specificity of the IHC staining was confirmed using the complete staining protocol on *Cthrc1* gene-deficient mouse skeletal tissues (**g**). An additional control is shown using *Cthrc1*-wildtype tissue sections that were incubated with secondary donkey anti-rabbit antibody alone, which produced no signal (**h**). CTHRC1+ cells were found in the arthritic pannus intimal lining (**a**, **e**, **f**) and at the bone-pannus and cartilage-pannus boundaries (**a**–**d**). *Dotted line* shows the border between cartilage (*Ct*) and bone (*Bn*) (**b**). *Fct* fibrocartilage, *Syn* synovial cavity. Sections were counterstained with alcian blue (**c**, **d**, **g**) or with hematoxylin (**a**, **b**, **e**, **f**, **h**). *Scale bars* are 100 μm (**a**), 50 μm (**b**, **c**, **e**, **g**, **h**), and 20 μm (**d**, **f**)

IHC staining revealed several sites of the protein location in arthritic synovium. CTHRC1 was expressed in cells located closer to bone, cartilage, and fibrocartilage surfaces (Fig. 3a–d). Intracellular protein expression was maximal in cells in close contact with the bone and gradually decreased towards the synovial cavity side of the pannus (Fig. 3b–d). A similar pattern was observed in the knee joints and in smaller joints of the hind paws. The intimal lining layer of the pannus that faced synovial cavities was frequently CTHRC1-positive (Fig. 3a, e, f). Some deposition of the protein was detected on the bone surface (Fig. 3c, d), in underlying synovial membrane connective tissue (Fig. 3e), and in the areas adjacent to the bone/cartilage surface.

Specificity of the IHC staining was confirmed using skeletal tissues of the *Cthrc1*-deficient mice generated earlier [20]. Tissue sections were stained using primary and secondary antibodies, and the complete IHC staining was negative (Fig. 3g). Similarly, negative staining was achieved when primary anti-CTHRC1 antibodies Vli-55 were substituted with normal donkey serum (Fig. 3h). IHC staining showed a similar pattern for synovial tissues of two different genetic backgrounds of the inbred BALB/c and (FVB/N × BALB/c)F1 hybrids.

### Co-localization of cells expressing synoviocyte-specific markers

**Fig. 4:**
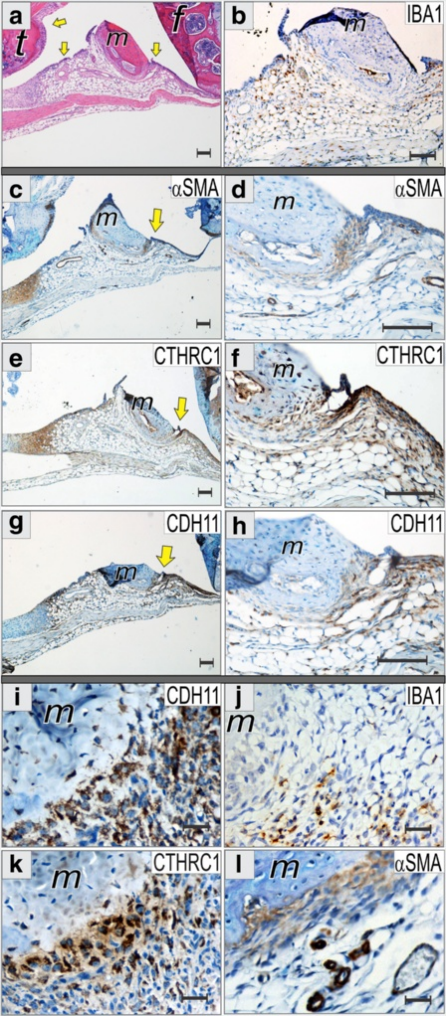
Co-localization of cells expressing synovial markers. Sagittal plane histological sections of the medial aspect of inflamed knee joints were stained with hematoxylin-eosin (**a**), macrophage-specific calcium-binding protein *IBA1* (**b**, **j**), alpha smooth muscle actin (*α-SMA*) (**c**, **d**, **l**), collagen triple helix repeat containing 1 (*CTHRC1*) (**e**, **f**, **k**), cadherin CDH11 (**g**–**i**). Sections were counterstained with hematoxylin or alcian blue. Meniscus (*m*) was used to align images; femur (*f*), tibia (*t*). Area of the pannus-meniscus junction labeled with *yellow arrowheads* (**c**, **e**, **g**) are zoomed in at panels **d**, **f**, and **h**. Representative immunohistochemical (IHC) images at panels **i**–**l** show the area of pannus-meniscus junction at higher × 40 objective magnification. At this structure, cells positive for CDH11, CTHRC1 and α-SMA IHC staining demonstrated a similar gradient distribution with maximal signal at the meniscus fibrocartilage-pannus border, while macrophage-specific IBA1 showed dispersed localization. *Scale bars* are 100 μm (**a**–**h**) and 25 μm (**i**–**l**)

CTHRC1-positive synoviocytes exhibited fibroblast-like morphology and were located at the sites that are characteristic for activated FLS (Figs. 1f, 3c–f). We further studied localization of cells expressing CTHRC1 and their co-localization with fibroblast, myofibroblast, and macrophage markers. For this purpose, we used median sagittal histological sections of the knee (Fig. 4). Despite the fact that larger knee joints are not the primary target of arthritis in either human patients or mice, multicellular pannus was easy to find within knee joint synovium upon routine histological examination (Fig. 4a, yellow arrowheads). We used the meniscus as a landmark to align and compare images (Fig. 4).

IBA1 is a calcium binding adaptor molecule also known as allograft inflammatory factor 1 that is involved in membrane ruffling and phagocytosis [21]. IBA1 is expressed in activated macrophages and also in inflamed synovium [22]. In mice with CAIA, IBA1+ macrophages were infiltrating the entire pannus, but were more abundant in synovial lining sides adjoining the synovial cavity (intimal lining), and were basically absent at the area of the bone-pannus interface (Fig. 4b, j). The size of the IBA1+ cells was much smaller than large CTHRC1+ fibroblast-like cells. Dispersed IBA1+ staining contrasted with CTHRC1 signal distribution.

Synovium was stained for α-SMA, which is expressed in vascular smooth muscle cells and in myofibroblasts. Expression of α-SMA is increased in the transition of normal FLS to activated FLS [23]. In our experiments, anti-α-SMA antibodies stained smooth muscle cells of the vasculature of the naïve non-inflamed synovium (data not shown), but IHC staining of synovial lining and pannus was found only in CAIA (Fig. 4c, d, yellow arrowhead). α-SMA+ cells were found mostly at the synovial intimal lining (Fig. 4c, d), and also at sites of the pannus contacting fibrocartilage of the meniscus (Fig. 4l). Vasculature was strongly positive for α-SMA (Fig. 4l).

CDH11 was previously established as one of the most specific markers for activated FLS [2, 6]. In this CAIA model, staining for CDH11 was most prominent in the intimal lining (Fig. 4g, h), although any part of the pannus, including the middle fibrous-like layer and the bone-pannus interface contained CDH11+ cells (Fig. 4i).

CTHRC1 IHC staining was found at the intimal lining and the bone-pannus interface, locations that greatly overlapped with sites of α-SMA and CDH11 expression (Fig. 4c-l). Taking together, we found CTHRC1 protein staining at several locations within the arthritic synovium. The strongest signal was found in inflamed pannus with some accumulation at the pannus borders represented by the intimal lining and the bone/cartilage–pannus interface. A much weaker and infrequent signal was found in meniscus fibrocartilaginous cells in contact with the pannus. Sometimes CTHRC1 positivity was found in ligaments and striated muscles, which could be remodeled due to inflammation. Practically no IHC staining was found in bone, articular cartilage, or growth plate chondrocytes (Figs. 1, 3, 4).

In brief, the conclusion of CTHRC1 synthesis by arthritic pannus FLS is supported by (1) fibroblast-like morphology of CTHRC1+ cells; (2) significant overlap between CTHRC1+, CDH11+ and α-SMA+ staining; and (3) no overlap with IBA1+ cell locations.

### CTHRC1 promoted motility of embryonic fibroblasts and RA-FLS

Overexpression of *Cthrc1* transgene promoted migration of cells from several lineages, including primary mouse embryonic fibroblasts, smooth muscle and cancer cells [13, 14]. The effect of CTHRC1 upon the cell motility of activated synoviocytes and the more general effect in inflammatory conditions have never been studied.

**Fig. 5:**
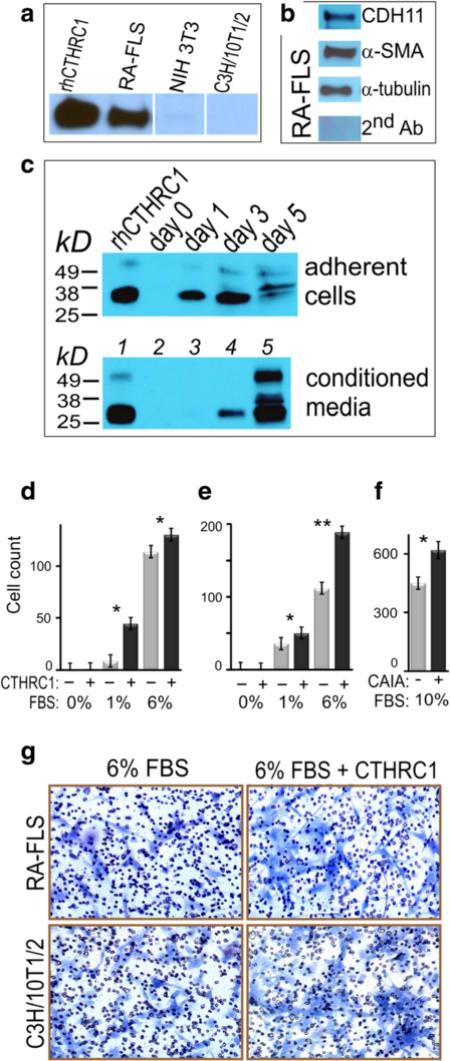
Collagen triple helix repeat containing 1 (*CTHRC1*) effects transwell cell migration. **(a)** Western immunoblotting detection of CTHRC1 in rheumatoid arthritis-fibroblast-like synoviocytes (*RA-FLS*), NIH 3T3, C3H/10 T1/2. RA-FLS express CTHRC1, but murine fibroblasts are negative for CTHRC1. **(b)** RA-FLS also express cadherin CDH11, alpha smooth muscle actin (*α-SMA*), and α-tubulin. Staining membrane with secondary antibodies alone was negative. **(c)** CTHRC1 is secreted into conditioned media upon transfection and overexpression in Chinese hamster ovary (CHO) cells. *Lanes*: (1) positive control 10 ng rhCTHRC1; (2) day 0; (3) day 1; (4) day 3; (5) day 5. **(d)** RA-FLS were treated directly in transwell chambers with rhCTHRC1 protein, while FBS concentration was varied from 0 %, 1–6 %. Cells that migrated through the membrane were stained with toluidine blue and counted under the microscope. *Solid black columns* represent cells treated with rhCTHRC1, *gray columns* are untreated cells. **(e)** murine C3H/10T1/2 fibroblasts were treated with rhCTHRC1 similarly to RA-FLS. **(f)** Serum from mice with acute CAIA (5 % serum mixed with 5 % FBS in complete media) showed the strongest promigratory effect. **(g)** Toluidine blue staining and microscopy of cells migrated through the membrane and spread on its opposite side after the completion of the experiment. *Numerous dots* are membrane pores of 8 μm in size. Data are representative of three similar but independent experiments. Statistical significance was calculated using Student’s *t* test: *p < 0.05; **p < 0.01 for treatment versus no treatment comparison

We examined expression of CTHRC1 in murine fibroblasts and in RA-FLS using western immunoblotting. Two lines of mouse embryonic fibroblasts NIH 3T3 and C3H/10T1/2 were studied, and both were negative for CTHRC1 production (Fig. 5a). On the contrary, RA-FLS were positive for CTHRC1 expression, and for cadherin CDH11 and α-SMA production (Fig. 5b). This staining of human synoviocytes paralleled IHC staining of the mouse CAIA pannus (Fig. 4).

At IHC staining of inflamed synovial joints, some deposition of CTHRC1 into bony and fibrocartilage tissues was noticeable (Figs. 3, 4). Expression of CTHRC1 by transiently transfected CHO cells in vitro and secretion of the protein into media was confirmed (Fig. 5c). Recombinant human rhCTHRC1 is expressed intracellularly in adherent cells with a peak at day 3 post transfection. After centrifugation of the conditioned media at 1000 × g for 15 minutes, protein was detectable in the media supernatant as a single protein band at day 3 (Fig. 5c). CTHRC1 continued to accumulate in the media until at least day 5, and there was no proteolytic degradation of the secreted protein.

To study whether CTHRC1 has a promigratory effect upon activated FLS and embryonic fibroblasts, we treated cells with purified recombinant protein and assayed cell motility using transwell plates. Neither RA-FLS nor C3H/10T1/2 fibroblasts migrated in the absence of serum even if CTHRC1 was provided (Fig. 5d, e), hence, the CTHRC1 required serum components for promoting cell motility. Cells were able to move through the membrane in presence of 1–6 % FBS, and CTHRC1 demonstrated a positive effect on transwell cell motility (1.4–5-fold, *p* < 0.05) (Fig. 5d, e). As a positive control, we used serum taken from mice with CAIA. A mixture of equal parts of arthritic serum and FBS (5 % arthritic serum and 5 % FBS) in complete media showed the strongest promigratory effect (Fig. 5f). At the end of the experiment, transmigrated fibroblasts had a normal morphological spread (Fig. 5g).

**Fig. 6:**
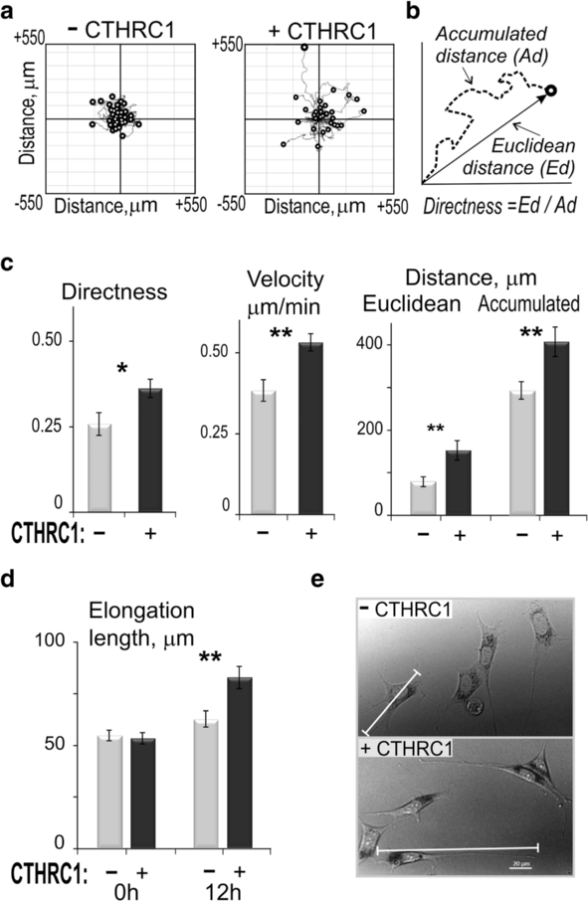
Time-lapse microscopy analysis of fibroblast migration under collagen triple helix repeat containing 1 (*CTHRC1*) treatment. NIH 3T3 fibroblasts seeded onto μ-slides were treated with rhCTHRC1 at 1000 ng/ml and observed using time-lapse microscopy. **(a)** Cell trajectories derived from stacks of images taken every 15 minutes during migration were presented as Rose plots to compare cell motility parameters. **(b)** Each cell trajectory was analyzed for accumulated distance (*Ad*), Euclidean distance (*Ed*), directness (*Ed/Ad*), and cell velocity (*Ad/time*). **(c)** Average values for 20–40 cells for each condition were compared. *Solid black columns* represent cells treated with rhCTHRC1, *gray columns* represent untreated cells. **(d)** Cell length was defined as a Euclidean distance between the most distant points, usually between the front of lamellipodia and the end of cell tail. Cell length is shown by a *white bar*. Phase contrast images of cells in the beginning and after 12 hours CTHRC1 treatment are presented. *Scale bar* is 20 μm. **(e)** Cell length was measured at the beginning and the end of observation period. Data represent four similar but independent experiments. Statistical significance was calculated using Student’s *t* test: *p < 0.05; **p < 0.01 for comparison of treatment versus no treatment

To study detailed cellular mechanisms of motility promoted by CTHRC1, we used time-lapse microscopy of NIH 3T3 fibroblasts in specially designed μ-slide chambers (IBIDI USA Inc., Madison, WI, USA). The μ-slide bridge was coated with collagen type I. We treated fibroblasts with rhCTHRC1 at 200–1000 ng/ml in 10 % FBS and monitored cells at the bridge using time-lapse microscopy for up to 14 hours. In these conditions that were similar to those in the transwell assay experiment, CTHRC1 also demonstrated a statistically significant promigratory effect (Fig. 6). We traced trajectories of individual cells and combined them in Rose plots (Fig. 6a). In the presence of CTHRC1, fibroblasts migrated a notably longer distance on average, although the effect varied greatly for individual cells. The velocity of cell movement, directness and accumulated distance traveled by cells all increased by 40 % (*p* < 0.01). The Euclidean distance increased by 93 % (*p* < 0.05), which indicated that in the presence of CTHRC1, fibroblasts were more prone to maintaining a specific direction of migration once it was selected by the cell.

Another clear effect of the treatment was the elongation of cells or increase of cell size along the front-tail axis. Cell size was measured between the front of the lamellipodium and the end of the cell tail (Fig. 6e). Upon rhCTHRC1 treatment, NIH 3T3 fibroblast cell size was significantly increased at 32 %, *p* < 0.01 (Fig. 6d, e). The effect of cell elongation was obtained due to the protraction of the cell tail.

## Discussion

In this study, we discovered that CTHRC1, otherwise a recognized marker of enhanced cancer metastases, is overexpressed in inflammatory conditions of murine arthritis. Therefore, we corroborated our earlier transcriptome-based finding that *Cthrc1* mRNA is highly inducible in CAIA inflammation in synovial joints, and CTHRC1 protein content increases more than 10-fold within 3 days from a basically undetectable level [9]. Due to clear inducibility in arthritis, specificity for the arthritic pannus, and the ability to be secreted into circulation, CTHRC1 could be a promising RA marker. In this study, the first pilot cohort of patients with RA had significantly increased circulating CTHRC1 (Fig. 2c), and high CTHRC1 correlated with increased CRP and with aggravated arthritis (Fig. 2d, e). Interestingly, plasma CTHRC1 did not increase in patients with solid tumors, where CTHRC1 is also strongly expressed [7, 8, 16]. This discrepancy indicates that ways of regulating CTHRC1 in the circulation might depend on tissue vascularization, protein stability, and different/other sites of protein production.

In synovium, CTHRC1 expression was detected at multiple locations, while the major positive tissue was pannus. Ligaments, muscles, and fibrocartilage were sometimes positive for immunostaining, but more studies are needed to understand why these sites are affected. In general CTHRC1 is a marker of tissue remodeling, and inflammation or wounding could spark its expression. The deposition of CTHRC1 in bone and fibrocartilage at the sites of their contact with pannus could be remnants of dead cells or could represent protrusions of FLS into bone. Synovial sites occupied with CTHRC1+ cells notably overlap with the distribution of α-SMA+ cells. This overlap was detected at the tissue (Fig. 4) and at the cellular levels (Fig. 5b). Expression of α-SMA is a characteristic feature of stromal cells of different origin. In the recent study of solid tumors, it was shown that CTHRC1 is produced by a tumor stroma [15]. Activated FLS are similar to stromal cells in their multipotency, i.e., the ability to differentiate into chondrogenic and osteogenic lineages [1].

Because of the clear correlation between the degree of inflammation, and CTHRC1 mRNA and protein levels and because the protein is expressed in fibroblast-like cells, our first hypothesis was that CTHRC1 expression promotes bone and cartilage erosion by activated FLS. Nonetheless, we did not observe co-localization of FLS-CTHRC1+ with sites of bone erosions. Also, CTHRC1+ cells comprised an intriguingly substantial portion of the pannus (Fig. 1). An alternative hypothesis is that CTHRC1 is labeling a remodeling tissue and mesenchymal progenitor cells that influx into the arthritic synovium to fight inflammation. In the CAIA murine model, it is particularly obvious that this new pathologic tissue of the pannus could not be formed due to the proliferation of normal FLS forming normal synovial membrane. In CAIA, hypercellular pannus is formed within 2–3 days after induction. Therefore, CTHRC1+ FLS (or their progenitors) have enough time only to migrate into the synovial cavity and self-organize into new tissue within the time frame of several days. In line with this hypothesis, CTHRC1 was shown earlier to be a marker for mesenchymal lineage cells, including osteoblasts and RA-FLS [24]. The presence of mesenchymal stem/stromal cells within the synovium has been demonstrated repeatedly [25–28].

Attempts to convert dermal fibroblasts or OA-FLS into RA-FLS using stimulation with IL-1β or a mixture of pro-inflammatory cytokines under hypoxia/normoxia were not successful [29], which suggests that the pro-inflammatory milieu is not sufficient to induce the de-differentiation of FLS in arthritic joints, and normal FLS and RA-FLS are somewhat different cell lineages. In this study, we found abundant amounts of CTHRC1 in inflamed synovium, which underlines the connection between inflammation and stromal cell activation, although more research is needed to prove that CTHRC1 is specific to synovial stromal cells and to determine cellular targets of the pro-motile activity of the protein. CTHRC1 increases cell motility, and therefore promotes the influx of cells into inflamed joints and formation of the multicellular pannus.

The effect of CTHRC1 upon cell motility is manifold and is not completely understood. Reports describe co-precipitation of CTHRC1 with components of non-canonical WNT signaling and stimulation of RhoA and Rac1 phosphorylation [30, 31]. In hepatocellular carcinoma CTHRC1 promotes cell adhesion through activation of integrin β1 and phosphorylation of focal adhesion kinase (FAK), while the activation of RhoA, but not Rac1 or Cdc42 plays a crucial role [32].

In this study, we demonstrated a positive effect of CTHRC1 upon motility of embryonic fibroblasts and RA-FLS. Interestingly, the protein regulated cell motility in two ways: both directness and velocity. The effect upon directness indicates that CTHRC1 induces cell polarization via recruiting potential CTHRC1 sensors/receptors at the leading edge of the lamellipodium. This effect of CTHRC1 might be connected with the redistribution of WNT signaling receptors and cell polarization (see also Fig. 6). The effect upon velocity of movement is different from the regulation of directness. The velocity effect should involve the regulation of focal adhesion formation/disassembly and stress fibers, and this is in line with CTHRC1 regulation of FAK and RhoA.

At this point, it is not clear whether CTHRC1 acts from cytoplasm or via surface receptors, or both. CTHRC1 siRNA approaches have been successfully used to deregulate cell motility, but other studies, including this one, effectively used exogenous CTHRC1 protein. The effect of the addition of rhCTHRC1 into media with murine fibroblasts lacking endogenous CTHRC1 suggests the presence of surface receptors. In contrast, in RA-FLS, which produce CTHRC1 endogenously, the mechanism of cell motility regulation could use both intracellular and extracelluar pathways.

Another possible mechanism of the regulation of cell motility might involve the Gly-X-Y motif in CTHRC1 protein. This motif is actually a ligand involved in cell adhesion by binding to α2β1 integrin [33–35]. Triple helix motif-containing proteins, including CTHRC1, might compete with collagens for cell adhesion receptors and therefore attenuate cell adhesion onto the bone surface, and, therefore, modulate cell motility. Recently, a collagen-mimetic triple helical supramolecule containing the integrin-binding sequence was used to interfere with α2β1 integrin-dependent cell adhesion [33]. Similar approaches might have potential for intra-articular interventions to control pannus activity and to treat arthritis.

## Conclusions

CTHRC1 was found in activated fibroblast-like synoviocytes in the inflamed synovium in murine arthritis and in plasma from patients with RA. CTHRC1 is produced by RA-FLS and increases its motility using an autocrine loop, but the protein also has promigratory potential upon other cells including fibroblasts that are not terminally differentiated. CTHRC1 promoted fibroblast polarization along the front-tail axis and increased the speed of migration and directness of cell movement. This promigratory effect on fibroblast-like synoviocytes is one of the mechanisms in the hypercellularity of arthritic pannus.

## Abbreviations

Ad, accumulated distance; BSA, bovine serum albumin; CAIA, collagen antibody-induced arthritis; CDH11, cadherin 11; CDNA, complementary DNA; CRP, C-reactive protein; CTHRC1, collagen triple helix repeat containing 1; DAB, 3,3′-diaminobenzidine; DAS28, 28-joint count disease activity score; DMEM, Dulbecco’s modified Eagle’s medium; Ed, Euclidean distance; ELISA, enzyme-linked immunosorbent assay; FAK, focal adhesion kinase; FBS, fetal bovine serum; FLS, fibroblast-like synoviocytes; Gapdh, glyceraldehyde 3-phosphate dehydrogenase; HRP, horseradish peroxidase; IACUC, Institutional Animal Care and Use Committee; IHC, immunohistochemistry; IL, interleukin; i.p., intraperitoneal; LPS, lipopolysaccharide; PBS, phosphate-buffered saline; PBS-BT, phosphate-buffered saline containing 0.1 % bovine serum albumin and 0.1 % Tween 20; siRNA, small interfering RNA; α-SMA, alpha smooth muscle actin

## Additional file

Additional file 1: CTHRC1 and cadherin CDH11 expression in synovium in early and later arthritis. Sagittal plane histological sections of the medial aspect of inflamed knee joints were IHC stained for CTHRC1 (**A**, **B**) and for cadherin CDH11 (**C**, **D**). Sections were prepared from arthritic joints at day 4 (**A**, **C**) and 14 (**B**, **D**) of arthritis development. Histological sections of the arthritic pannus-meniscal junction and meniscus (*m*) are shown. Sections were counterstained with hematoxylin. (PDF 1154 kb)
