# Supplementary figures and images for: Collagen triple helix repeat containing 1 is a new promigratory marker of arthritic pannus
Source: Arthritis Res Ther. 2016 Jul 19;18:171. doi: 10.1186/s13075-016-1067-1 (PMC4950773; doi:10.1186/s13075-016-1067-1)

Additional File-1  
Figure

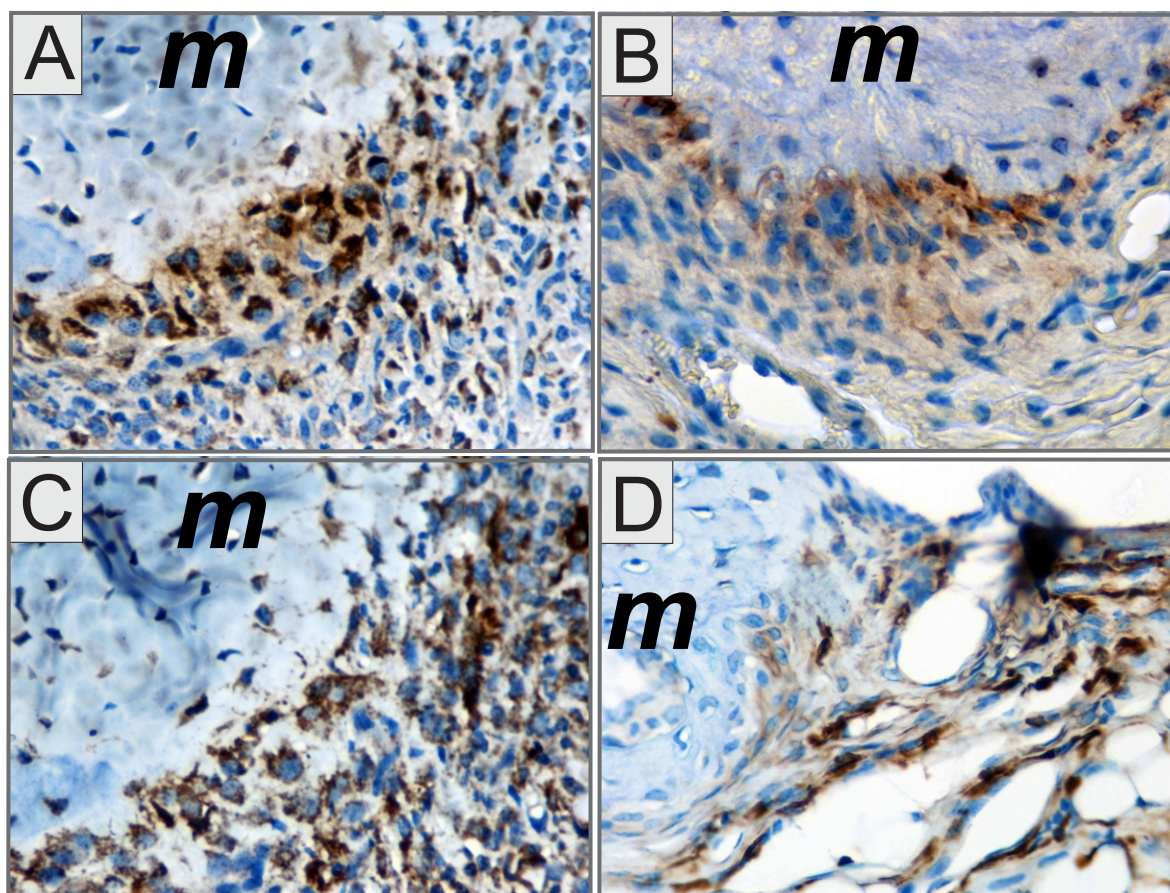

Supplement: Additional file 1: — CTHRC1 and cadherin CDH11 expression in synovium in early and later arthritis. Sagittal plane histological sections of the medial aspect of inflamed knee joints were IHC stained for CTHRC1 (A, B) and for cadherin CDH11 (C, D). Sections were prepared from arthritic joints at day 4 (A, C) and 14 (B, D) of arthritis development. Histological sections of the arthritic pannus-meniscal junction and meniscus (m) are shown. Sections were counterstained with hematoxylin. (PDF 1154 kb) [file 13075_2016_1067_MOESM1_ESM.pdf]
